# From athletic excellence to academic influence: a study of retired Chinese athletes transitioning into the higher education sector

**DOI:** 10.3389/fpsyg.2024.1401575

**Published:** 2024-06-18

**Authors:** Yutao Zhou, Zhiming Zhang

**Affiliations:** ^1^Colleges of Physical Education, Hunan University of Technology, Zhuzhou, China; ^2^Hunan Research Centre for Excellence in Fitness, Health, and Performance, Zhuzhou, China

**Keywords:** retired athlete, career transition, higher education, challenges, barriers

## Abstract

While the career transition of athletes has been explored to several extents, it is often marked by complex psychosocial challenges and requires a redefined sense of identity and professional purpose. Research to date has predominately focused on the disadvantages or inequity emerging from broad social demographics without delving into specific career transition pathways, such as into higher education settings. This study specifically investigates the unique psychosocial factors underlying the career transition of retired Chinese athletes into higher education. We conducted an interview phase with retired Chinese athletes (Phase 1, *n* = 17) and a Delphi phase with senior human resources (HR) managers from Chinese higher education settings (Phase 2, *n* = 15). This approach allowed us to understand the lived experiences and challenges of these athletes within the Chinese cultural and social context, as well as HR experts’ perceptions of their career transitions, respectively. The results unveil crucial psychosocial factors that motivate, and the barriers that challenge, and difficulties retired Chinese athletes in their transition, informing intervention and policy efforts to facilitate their successful integration into higher education.

## 1 Introduction

The career transition of athletes has been recognized as a complex and multifaceted process. It involves various dimensions, such as athletic (Hong and Minikin, 2023), psychological (Oulevey et al., 2024), academic (Henriksen et al., 2023), and vocational aspects (Schmid et al., 2023). Terminating an athletic career is viewed as a transitional process influenced by athletes’ identity and developmental experience rather than a singular event (Wylleman et al., 2004). As such, it is critical to tackle psychosocial factors associated with issues emerging at athletes’ career termination and address them to facilitate a healthy and smooth career transition.

The focus within the literature has been on the role of identity in the career transition processes, identifying the challenges athletes face as they develop their post-sport careers (Cosh et al., 2013). Steele et al. (2020) and Murphy et al. (1996) highlighted the significance of athletic identity in career maturity among athletes, stressing the need to address identity issues when assisting athletes in career transitions. This identity does not simply vanish upon retirement; rather, it evolves as athletes seek new roles that allow them to remain connected to their sports legacy (Dimoula, 2013; Wendling and Sagas, 2021). Also, the quality of post-sport career transitions is associated with pre-retirement preparation, such as education and financial support (Robnik et al., 2022). Several studies have explored the dual career paths of student-athletes, examining their engagement with university majors, plans for dual careers, and the demands encountered during such transitions (Condello et al., 2019; Vickers and Morris, 2022). This line of inquiry extends to employed Olympic athletes, providing insights into dual career support and its effect on the quality of transitions (Robnik et al., 2022). Recent research has also highlighted the importance of considering specific demographic variables, such as gender, ethnicity, and socioeconomic background, influencing how different groups experience and manage their transitions into new career roles (Carapinheira et al., 2018). For instance, studies focusing on female athletes have uncovered the challenges posed by societal norms and the impact of cultural factors on their transition experiences (Cartigny et al., 2023; Zhu, 2023). Despite the growing literature, there remains a need for a more comprehensive approach to address the varied and often crisis-laden transitions faced by athletes, especially those involving specific career settings and shifts in identity (Wylleman et al., 2004; Surujlal and Zyl, 2014; Brown et al., 2015). The literature calls for tailored support mechanisms to facilitate smoother transitions into specific career pathways, ensuring more successful post-athletic career adjustments.

Meanwhile, factors that precede athletes’ challenges in career transition have been well-documented in the literature. For instance, the quality of post-sport career transition has been associated with factors such as pre-education and support-related finances (Robnik et al., 2022). Also, the nature of career termination, whether voluntary or involuntary, has been identified as a factor influencing the ease of transition (Carapinheira et al., 2018). Therefore, Wylleman et al. (2004) proposed a “beginning-to-end” approach to understanding the challenges and issues in athletes’ career transitions. More specifically, these researchers posited that the career transition-related problems faced by athletes are associated with their life-span experience across individual, psychosocial, and academic or vocational levels, for example. Many of these issues in athletes (e.g., insufficient life skills, lack of education) would inflate the perceived pressure and stress in athletes under a career transition process, leading to possible traumatic experiences. Such a viewpoint has received empirical support from various sources (Carapinheira et al., 2018; Condello et al., 2019; Robnik et al., 2022; Vickers and Morris, 2022).

However, current knowledge athletes’ career transition rarely delves into the specific pathways and the unique psychosocial factors of pursuing a particular career pathway after retiring from or terminating a competitive athletic career in literature, especially the specific transition into teaching careers or higher education teaching positions. Indeed, recent research has unveiled an increasing trend and interest among retired athletes seeking careers in various educational levels (Iio et al., 2023). Teaching in the higher education setting has become a considerably popular second career among elite athletes, because such a transition allowed them to utilize their expertise and generate impacts on shaping the next generation via higher education (Iio et al., 2023). Similar findings were also documented in retired athletes who used to compete at the Olympic level (Lin et al., 2016). Nevertheless, research to date has yet to receive much attention on the psychosocial hurdles that retired athletes face in the career transition into higher education. Therefore, the primary aim of the current study was to understand the unique psychosocial hurdles influencing athletes’ career transitions into higher education settings, specifically focusing on the experiences of retired athletes with a Chinese background. This study explores their motivations for entering higher education, as well as the barriers, difficulties, and challenges they encounter as different aspects of the psychosocial hurdles faced by retired athletes in their career transition.

## 2 Methods

### 2.1 Research design

To explore the lived experience of retired athletes in their transition into a career in higher education, we conducted a series of semi-structured interviews with retired athletes working (*n* = 8) or expected to be working (*n* = 9) in a higher education institution (Phase 1). However, relying purely on retired athletes’ lived experience in understanding their career transition into higher education can be prone to recall and participant bias. To further understand the common issues for athletes’ career transition into the higher education settings and how athletes’ lived experience may account for these issues, we conducted a three-round Delphi study involving a group of senior human resources managers (*n* = 15) who had experience of recruiting retired athletes (Phase 2). Such an approach provided an ‘informant’ perspective to retired athletes lived experience and thus prevented potential bias due to self-report and single-source (i.e., athletes) data. We synthesized the findings from both phases to generate new knowledge and inform future career development programs facilitating athletes’ career transition (Table 1).

**Table 1:** Details of target participants.

	Participants	Recruitment condition and number	Experience limitation	Inclusion criteria
Phase 1	Athletic	Currently working in higher education (8, Male 4, Female 4)	Working experience (More Than 2 Year)	Over 10 years of professional sports experience in China
		Currently seeking job opportunity in higher education (9, Male 4, Female 5)	Seeking experience (More than 2 year)	Over 10 years of professional sports experience in China
Phase 2	Experts	Seven different universities in China (15, Male 10, Female 5)	Senior human resources managers	Over 10 years of recruitment experience in China

### 2.2 Participants

We recruited 17 retired national-level athletes (Phase 1) and 15 senior human resources (hereafter referred to as “experts”) managers (Phase 2) from seven different universities in China. Phase 1 participants, all of whom had over 10 years of professional sports experience and were aged between 26–37 years old, represented a diverse range of sports disciplines and competition achievements. These included Olympic Game Champions, Asian Game Champions, National Game Champions, and Provincial Game Champions in sports such as Badminton, Table Tennis, Tennis, Artistic Gymnastics, Gymnastics, Sports Dance at the Asian Games, Swimming, and Athletics. Detailed participant information, including their sports discipline and highest achieved rank in their respective fields, is provided in Table 2.

**Table 2:** Sporting experience of the Phase 1 athletic participants.

Phase 1: Working in higher education		Phase 1: Seeking job opportunity in higher education	
W1 (AGC+SD+A28)	W6 (NGC+AL+A31)	S1 (NGC+AL+A27)	S6 (PGC+AL+A26)
W2 (NGC+T+A34)	W7 (NGC+AL+A34)	S2 (AGC+SD+A28)	S7 (PGC+AL+A29)
W3 (PGC+BM+A30)	W8 (PGC+S+A33)	S3 (PGC+TT+A33)	S8 (NGC+AL+A26)
W4 (PGC+G+A34)		S4 (NGC+AG+A32)	S9 (AGC+G+A30)
W5 (OGC+BM+A37)		S5 (PGC+S+A27)	

OGC, Olympic Game Champion; AGC, Asia Game Champion; NGC, National Game Champion; PGC, Provincial Game Champion; BM, Badminton; TT, Table Tennis; T, Tennis; AG, Artistic Gymnastics; G, Gymnastics; SD, Sports Dance-Asian Games; S, Swimming; AL, Athletics; A, Age.

These participants were either working (*n* = 8; males = 4, females = 4; average working experience in higher education = 3.5 years) or seeking job opportunities (*n* = 9; males = 4, females = 5; average job-seeking duration = 2 years) in higher education institutions. The age range for those employed was 28–37 years, while for those seeking employment, it was 26–33 years. Phase 2 consisted of senior human resources managers (*n* = 15; males = 10, females = 5) with over 10 years of experience in academic staff recruitment, representing seven different universities across China.

### 2.3 Procedures

Following ethics approval from the lead author’s university, Phase 1 involved recruiting retired athletes using convenience and snowball sampling methods (Robinson, 2014). Information about the study was disseminated through networks, including coaches and higher education institutions. Interested athletes contacted the lead author for more details. After providing informed consent and completing a demographic questionnaire, participants engaged in semi-structured interviews designed to explore their experiences transitioning into higher education careers. The purpose of these interviews was to gather in-depth insights into the challenges and opportunities these athletes faced during their career shifts. Specific areas covered included their initial decision to pursue higher education and the barriers, challenges, and difficulties experienced during their transition. These interviews, conducted by the second author, who holds a doctorate and is experienced in qualitative research, typically lasted between 45 to 60 min. The interviews were structured around key themes such as initial career transition challenges and the impact of their athletic background on their current roles. All interviews were conducted in a confidential manner (i.e., no observer attending the interview, no third party other than the research team and the participant knew the interview was taking place) and were audio recorded for transcription before data analysis. Each participant was given a pseudonym for the audio recording and transcription without any personally identifiable information collected or stored.

For the Phase 2, 15 experts were recruited via an identical approach as in Phase 1. The purpose of these semi-structured interviews was to reach a consensus on the significant hurdles retired athletes faced when transitioning into teaching roles in higher education. This involved the following rounds: Round 1, (Step 1) Experts were recruited for the panel; (Step 2) Participants engaged in semi-structured interviews where they were encouraged to discuss at length the significant hurdles retired athletes encountered when transitioning into teaching roles in higher education. These interviews were conducted face-to-face and verbally, and the discussion was guided by the interviewer based on a series of pre-determined open-ended questions with appropriate follow-ups dependent on each participant’s response to the question (thus semi-structured). To ensure accuracy in capturing their responses, all interviews were audio-recorded for further transcription and analysis. Subsequently, the initial themes identified through inductive thematic analysis were shared with the experts to gather their initial feedback and additional insights. (Step 3) An inductive content analysis was conducted to gain the sub-themes from raw data of Step 2; Round 2, (Step 4) Summarised sub-themes were sent to the expert panel for consensus and analysis of each sub-theme’s level of homogeneity and convergence (all sub-categories with a mode of 3 or greater were retained); Round 3 iterated step 4 until the panel reached consensus. The Delphi method prevented possible errors in face-to-face meetings via anonymity, an iterative structure, synchronicity, and controlled feedback (Di Zio and Maretti, 2014).

### 2.4 Inductive thematic analysis

We used Braun and Clarke (2006)‘s inductive thematic analysis (ITA) technique to analyze the Phase 1 interview data. ITA is a method for identifying and analyzing themes or patterns in transcribed subjective data (e.g., transcribed focus group scripts). According to Braun and Clarke (2006), the ITA procedure must not relate to any pre-existing theoretical framework for analyzing data, which was considered appropriate because the focus was on exploring data that reflects diverse experiences (Bernard et al., 2016). To increase the rigor of the analysis and reduce the potential bias of researchers’ prejudgment, we adopted an inductive approach to analyze the transcribed script at a semantic rather than explicit level (Mackieson et al., 2019). We followed ITA guidance, see Byrne (2022), and adopted the recommended six-step approach, including familiarizing data (i.e., Thoroughly reading and reviewing the entire dataset to develop a deep understanding of the information.), generating initial codes (i.e., Shorthand labels used to describe relevant information for research questions), generating themes (i.e., Understanding the overall meaning and significance of data by analyzing as a whole.”), reviewing themes and identifying possible sub-themes (i.e., conducting a recursive review of the candidate themes about the coded data items and the entire dataset), defining and naming themes (i.e., presenting a detailed analysis of the thematic framework), and producing the report (i.e., Using a recursive approach to report writing). After the second author transcribed audio-recorded data, each author coded all the transcripts separately by creating labels from the data. Each of the authors employed an iterative process to assign new codes when applicable, going backwards and forwards between the transcripts. Once codes had been assigned to sections of the transcripts, we separately created sub-themes by making connections between the discrete codes. Braun and Clarke (2006) stated a mismatch may occur here if inadequate coding has happened. Still, by even bracketing our views, our diverse experiences brought about different thematic identification. Finally, the authors met to discuss similarities, differences or conflicts through a collaborative approach where we each acted as critical associates to the others, and we agreed on several subthemes, which we collaboratively grouped into themes.

In analyzing Phase 1 and Phase 2 data, we employed NVivo software to assist with the coding and thematic analysis. This software enabled us to systematically organize and code the transcribed data, facilitating a rigorous and inductive approach to theme development. Additionally, to validate the authenticity of these findings, 10 participants (5 Working in Higher Education and 5 Seeking Job Opportunities) were invited to review the results, a process known as member checking. These participants were asked to evaluate the accuracy and resonance of the themes and sub-themes identified in the analysis. They provided feedback on whether these interpretations accurately reflected their experiences. Discrepancies in interpretations during the analysis phase were addressed by revisiting the original data and incorporating participants’ feedback. To ensure the reliability of our preliminary conclusions, two separate researchers deliberated over the identified dimensions and sub-themes until they reached a mutual agreement (Marshall and Rossman, 2014). The feedback from the participants generally supported the identified themes, enhancing the credibility of the findings.

### 2.5 Delphi analysis

The Delphi method, originally developed by the RAND in the 1950s (Sablatzky, 2022), is a structured communication technique designed to systematically gather expert opinions and achieve consensus on complex issues (Sablatzky, 2022). This iterative process involves multiple rounds, where feedback is provided anonymously to prevent any one opinion from dominating and to reduce potential bias (Quirke et al., 2023). The key characteristics of the Delphi Analysis include anonymity, iteration, controlled feedback, and statistical aggregation of group responses (Ismail and Taliep, 2023).

In Phase 2 of this study, following the initial inductive thematic analysis, this study engaged in a Delphi process to refine and validate the findings from the inductive thematic analysis and to further explore emerging new themes. The Delphi Analysis was chosen in this research for its robustness in achieving expert consensus and its effectiveness in addressing complex topics where opinions may diverge (Ismail and Taliep, 2023), which involved three rounds of surveys sent to a panel of experts who participated in the initial interviews (Quirke et al., 2023). In Round 1, the HR experts attended a semi-structured interview discussing their opinions about retired athletes’ hurdles in career transition into the HE setting. This led to the creation of a list of initial themes via thematic analysis of the interview transcription, as was done in Phase 1. In Round 2, we provided details about the Round 1 themes and asked the participating experts to rate the relevance and accuracy of the revised themes. Then, the rates were analyzed to assess the degree of homogeneity and convergence among the experts’ opinions. The (final) Round 3 involved a re-iteration of Round 2 results and process, of which the analysis of the final ratings was considered as consensus from the participating experts.

Importantly, in the Delphi process, all participating experts did not know each other, thus ensuring that the experts’ opinions were not influenced by others, thus preserving the integrity of the individual inputs. Between rounds, the study systematically collated and analyzed responses to identify patterns and shifts in opinions, guiding modifications to the themes. This iterative process validated our initial findings and enriched our understanding by incorporating diverse expert perspectives (Ismail and Taliep, 2023). The final themes were endorsed by all participating experts, indicating a strong consensus and adding a robust layer of validity to our study’s conclusions.

## 3 Results

### 3.1 Phase 1: retired athletes’ interview

In Phase 1, the research team identified 48 initial concepts/codes subdivided into 17 sub-categories. Following this, the sub-categories were integrated into 10 main categories and 4 main themes (see Table 3 for a summary, followed by detailed elaborations). To provide more context, the total word count of the interview transcripts amounted to approximately 75,000 words. Each interview transcript varied in length, ranging from 3,000 to 5,500 words. The development of the 48 initial concepts/codes was a result of an extensive coding process where all emerging themes were identified, capturing the full range of hurdles encountered by the interviewees, even if a theme appeared only once. These concepts/codes were determined through iterative coding sessions to ensure that they reflected the experiences and perspectives, preventing any themes from being overlooked in the iterative process.

**Table 3:** Themes, categories, subcategories, and representative citation of experiences from participants.

Themes	Main-categories	Sub-categories
Motivation	Passion and advocacy	Passion for sports and physical education
		Advocacy for physical education and health
	Personal identity	Seeking a sense of purpose and identity post-retirement
		Desire for a stable and fulfilling career post-sports
		Desire to give back and contribute to society
	Professional development and expertise utilization	Professional development and continuous learning
		Leveraging expertise and experiences
	Financial and stability considerations	Financial security and benefits
Barriers	Professional recognition and value	Status and recognition of the profession of physical education
		Identified a widespread undervaluation of physical education roles in higher education
	Systemic and institutional barriers	Lack of legal and regulatory safeguards
Difficulties	Education qualification	Mismatched educational qualifications
	Adaptation to educational environment	Development and changes in sports education
		Difficulties faced by higher education professionals due to audience diversity
Challenges	Educational and professional development	Lack of teaching experience and capabilities
	Psychological and identity-related adjustments	Challenges in psychological and physical adjustment
		Lack of professional identity and satisfaction

#### 3.1.1 Motivation

Exploring retired athletes’ transitions into the higher education sector revealed “Motivation” as a pivotal theme, encompassing various driving forces behind their career shifts. This theme is intricately divided into five main categories, each highlighting different facets of motivation: “Passion and Advocacy,” “Personal Identity,” “Professional Development and Expertise Utilization,” and “Financial and Stability Considerations.”

##### 3.1.1.1 Passion and advocacy

The transition of retired athletes into the higher education sector is deeply influenced by their enduring passion for sports and a strong sense of advocacy for physical education and health. This dual motivation reflects a desire not only to stay connected with their athletic roots but also to utilize their platform for broader societal impact.

###### 3.1.1.1.1 Passion for sports and physical education

The foundational element driving many participants toward academic roles is their deep-seated passion for sports. One participant said: *“Every class is a chance to turn a student into a sports lover, just like I am. It’s passing the torch in a way that keeps me close to the field.” (W3: Li)*. This expression highlights the intrinsic motivation to influence the next generation’s appreciation for sports and physical education, echoing a desire to extend their advantage beyond their athletic careers.

###### 3.1.1.1.2 Advocacy for physical education and health

Beyond a personal passion, the participants articulate a compelling vision for advocacy in physical education and health. One educator emphasized the mission-driven aspect of their role: “*It’s more than just a job; it’s a mission…make sure the next generation values their health and knows how to maintain it…”(W8: Jun)* Similarly, another participant reflected on their role as an advocate: *“I’m fighting the good fight, not in the ring anymore, but in the classroom. Promoting health, fitness, and some discipline, I learned as an athlete.”(S4: Kong).*

These expressions of passion and advocacy illustrate a significant motivational factor for retired athletes transitioning into the higher education sector. Their commitment to fostering an appreciation for sports and health among students not only serves as a continuation of their athletic legacy but also as a vital contribution to societal well-being.

##### 3.1.1.2 Personal identity

Exploring “Personal Identity” reveals the ways retired athletes seek new avenues for self-definition and purpose after their sports careers, which underscores the psychological transition from being recognized primarily for athletic achievements to embracing roles that offer personal and societal contributions.

###### 3.1.1.2.1 Seeking a sense of purpose and identity post-retirement

The shift toward academia is often driven by a search for meaning beyond athletic identity. Participants expressed a desire to rediscover themselves and their societal roles through teaching and mentoring. One vivid reflection illustrates this journey: *“It’s like, one day you are the star on the field, and the next, you are trying to figure out your next play in life. Teaching gives you that chance to be part of something bigger, to shape the future in a way that’s just as fulfilling as winning any championship.”(S7: Dong)*.

###### 3.1.1.2.2 The desire for a stable and fulfilling career post-sports

Stability and fulfillment emerge as critical considerations, with teaching providing a structured yet rewarding career path. One participant shared, *“Finding a new passion in teaching gave me the stability I sought after my sports career ended. It’s a different kind of fulfilment, knowing you are making a lasting impact.”(S9: Han)*.

###### 3.1.1.2.3 Desire to give back and contribute to society

The transition to academic roles reflects a broader desire to contribute to society. Another participant’s experience captures this sentiment: *“After retiring, I felt lost. But then I discovered teaching and realised I could use my experiences to help others. It’s more than sports; it’s about teaching life skills and making a difference.”(S3: Wu)*.

These reflections indicate the complex process of identity reformation among retired athletes. Transitioning to the higher education sector not only provides a platform for professional development but also facilitates a profound personal transformation, enabling individuals to forge new identities centered around mentorship, education, and societal contribution.

##### 3.1.1.3 Professional development and expertise utilization

The theme highlights how retired athletes leverage their unique skills and experiences to foster personal growth and contribute meaningfully to the academic sector, which encompasses continuously pursuing knowledge and applying their sports-related expertise in educational settings.

###### 3.1.1.3.1 Professional development and continuous learning

Participants emphasized the importance of ongoing learning and professional development in their new careers. One athlete-turned-educator reflected, *“Every day’s a school day, even for the teacher. Transitioning from the field to the classroom has been a continuous learning journey.”* Another shared, *“Getting into teaching was my next big league. It’s about pushing yourself to keep learning, just in a different arena.”(W7: Yue)*.

###### 3.1.1.3.2 Leveraging expertise and experiences

Bringing their wealth of experience from the sports world, retired athletes contribute a unique perspective to the academic field. *“Stepping into the classroom, I bring the field with me. It’s about using what I know and have lived to teach lessons beyond textbooks,”* remarked one participant. Another noted, *“I’ve got a lifetime of sports to share. It’s not just about the game, but the discipline, teamwork, and leadership that sports teach you.”(W5: Ling)*.

These quotes directly from the participants underscore the dual facets of this theme: the commitment to personal and professional development and the utilization of their sports expertise to enrich their roles in academia. This not only aids in their transition but also enhances the educational experiences of their students, bridging the gap between theoretical knowledge and real-world application.

##### 3.1.1.4 Financial and stability considerations

The theme reflects the pragmatic aspect of retired athletes’ motivation to transition into the higher education sector, which encompasses the desire for financial security and the benefits of academic positions, highlighting the importance of stable income and employment benefits in their career choices post-retirement.

###### 3.1.1.4.1 Financial security and benefits

Participants expressed a keen awareness of the financial stability and benefits of academic roles. One individual candidly shared, *“Let us be real, the paycheck and the stability that comes with a university job cannot be ignored. It’s about securing a future for me and my family.”(S5:Chang)*. This statement underscores the practical considerations influencing pursuing a career in academia beyond passion or identity. Another participant elaborated on the decision-making process, noting, “*Transitioning to teaching was also about finding a role where I could be financially stable and still feel like I’m making an impact. The benefits, like health insurance and retirement plans, are things you start to think about more as you age.”(W5: Ling)*.

These insights reveal that, alongside the intrinsic motivations of passion, identity, and the desire to impact society, financial and stability considerations play a crucial role in guiding retired athletes’ transition into academic careers. The emphasis on financial security and benefits highlights the multifaceted nature of their motivations, where practical concerns are balanced with the desire to continue contributing meaningfully to a new domain.

#### 3.1.2 Barriers

Within the exploration of retired athletes’ transitions into the higher education sector, the theme of “Barriers” emerges as a significant counterpoint to the motivational drivers, presenting the factors perceived by participants as hurdles that hinder their progress and are context-specific, insurmountable, and difficult to change or lack personal control. This theme is split into “Professional Recognition and Value” and “Systemic and Institutional Barriers.”

##### 3.1.2.1 Professional recognition and value

The theme shows the hurdles retired athletes face regarding societal and institutional perceptions of their new roles in the academic world, which is divided into two main concerns: the status and recognition of the physical education profession and the identified widespread undervaluation of physical education roles in higher education.

###### 3.1.2.1.1 Status and recognition of the physical education profession

Transitioning from celebrated athletic careers to academic roles, many retired athletes encounter a significant shift in the societal and educational system’s valuation of their new roles. One participant expressed this sentiment vividly*: “It’s like you have moved from being at the top of your game to a position where you have to justify your existence constantly. In the eyes of the world, and sometimes even within the schools themselves, physical education does not seem to hold the same weight as other subjects. You go from feeling like a hero to feeling like you are on the boundary, fighting for recognition and respect.”(W7: Yue)*.

###### 3.1.2.1.2 Identified a widespread undervaluation of physical education roles in higher education

This undervaluation extends beyond personal feelings of diminished status to a broader institutional and systemic issue. Participants noted a pervasive need for more support and recognition for physical education, impacting not just their sense of worth but also their ability to contribute effectively within their roles. *“The struggle is real. You’ve lived a life where your physical prowess was celebrated, but in academia, it’s a different ballgame. The widespread undervaluation of physical education roles means you are often fighting for resources, recognition, and even respect.”* Another participant added, *“It’s not just about fighting for your sense of worth; it’s about highlighting the value of physical education to the broader educational agenda. This lack of support and recognition affects not just us as individuals but the students we aim to inspire and educate.” (W3: Li)*.

These insights highlight the significant barriers related to professional recognition and value faced by retired athletes who transition into academic roles within physical education. The undervaluation of their professional contributions poses difficulties not only to their identity and sense of worth but also to the broader advocacy for physical education as a critical component of the educational curriculum.

##### 3.1.2.2 Systemic and institutional barriers

This category highlights the complexities of navigating educational institutions’ policies, practices, and cultures that may not always align with or support integrating professional athletes into academic roles.

###### 3.1.2.2.1 Lack of legal and regulatory safeguards

One of the key issues identified is the need for comprehensive legal and regulatory frameworks specifically designed to facilitate athletes’ transition into academia. Participants described feeling as though they were *“navigating a maze without a map,” underscoring the confusion and difficulties faced due to the lack of clear pathways and protections for athletes seeking to leverage their skills and experiences in educational settings. One educator remarked, “It’s like navigating a maze without a map. You know there’s a way through, but without clear safeguards and pathways, it’s all too easy to hit dead ends.”(S6: Zhou)*.

Furthermore, the issue extends beyond individual experiences to reflect a broader systemic oversight, as another participant highlighted: *“The lack of safeguards is not just a hurdle for us as individuals; it’s a reflection of how the system is not designed to recognise or accommodate the unique contributions we can make. It’s frustrating to see the potential for impactful educational contributions being stifled by bureaucratic inertia.”(W2: Ping)*.

These reflections point to a critical need for systemic changes within higher education institutions and regulatory bodies to better accommodate and value the unique perspectives and skill sets that retired athletes bring to the academic arena. The hurdles associated with navigating these systemic and institutional barriers not only impact the transition process for the athletes themselves but also limit the potential for enriching educational environments with diverse experiences and insights.

#### 3.1.3 Difficulties

The transition of retired athletes into the higher education sector has revealed “Difficulties” as a fundamental theme, which presents the factors that pose significant hurdles but are perception-based and thus more likely to be manageable and potentially overcome with effort, resources, or adaptation. These difficulties can be categorized into “Education qualification difficulties” and “Adaptation to educational environment difficulties.”

##### 3.1.3.1 Education qualification difficulties

The theme addresses the significant difficulties retired athletes face due to mismatches between their formal education levels and the qualifications required for teaching positions within the higher education sector. This gap is often a result of years dedicated to developing physical skills and achieving sports excellence, which may have limited their engagement with traditional educational pathways.

###### 3.1.3.1.1 Mismatched educational qualifications

Many retired athletes are disadvantaged when attempting to transition into academic roles, as their educational qualifications may not meet the necessary criteria for teaching positions, which is PhD level. Participants expressed their frustrations and the stark realization of these difficulties. One shared, *"Yeah, it's like, all those years focused solely on sports, aiming for gold, and suddenly, you're supposed to fit into this entirely different world… I mean, I've got medals, but when it comes to degrees? Not so much. And it hits you hard, you know? …”(S7:Dong).* Also, the feeling of being unprepared and undervalued despite a willingness to contribute to the academic realm was a common theme. *"It's frustrating, really. You've dedicated your life to excelling in one area, only to discover it's not enough. It's like… you're ready to give, teach, and inspire. But there are these difficulties, invisible yet so tangible, holding you back. Makes you wonder if it's even worth the leap, you know?" (W8:Jun)*.This theme vividly captures the tension between the desire to transition into educational roles and the difficulties related to educational qualifications that retired athletes face, highlighting the need for more inclusive and flexible pathways into the higher education sector for individuals with non-traditional educational backgrounds.

##### 3.1.3.2 Adaptation to educational environment difficulties

The theme highlights the need for adjustments in their approach to teaching and interaction within these new roles when interviewing retired athletes who are employed in higher education. This theme is broken down into two aspects: Further Development in Higher Education and the specific difficulties faced due to audience diversity in higher education.

###### 3.1.3.2.1 Further development in higher education

The transition from athlete to educator involves a significant shift in perspective and role. Participants noted the necessity of embracing a continuous learning mindset to adapt effectively. One described: *“It’s a whole new learning curve, really…Back then, I was the one performing, pushing my limits…”(W1:Ming).* Another emphasizes the adjustment process: *“You find yourself back in the role of a student, in a way…about adapting your teaching methods, being flexible, and always open to learning from the Environment.”(S4: Kong)*.

###### 3.1.3.2.2 Difficulties faced by higher education professionals due to audience diversity

This aspect highlights the difficulties arising from the diverse backgrounds and needs of students in higher education. Educators must develop strategies to engage effectively with this heterogeneity. A participant reflected on this: *"Every class feels like a mini society, with every student bringing their own stories, difficulties, and goals. It's about finding common ground, where you can reach out and connect with each of them, respecting their diversity while fostering a unified learning environment."(W4: Wei)*.These insights underline the multifaceted nature of the adaptation process for retired athletes entering the educational sector. Beyond adjusting to the role of an educator, there’s a critical need to navigate the evolving setting of higher education and to effectively engage with a diverse student body, each with unique expectations and learning styles.

#### 3.1.4 Challenges

In the examination of retired athletes’ transition into the higher education sector, the theme of “Challenges” stands out in contrast to the “Barriers” and “Difficulties,” which present the factors that encompass broader, ongoing hurdles requiring continuous effort and adaptation, potentially underpinned by opportunities. These challenges can be categorized as “Educational and professional development” and “Psychological and identity-related adjustments.”

##### 3.1.4.1 Educational and professional development

This theme encapsulates the challenge individuals face as they navigate the shift from athletic to educational environments, highlighting the need for formal teaching qualifications and the development of new professional capabilities.

###### 3.1.4.1.1 Lack of teaching experience and capabilities

One of the significant challenges identified is the need for formal teaching experiences and capabilities among retired athletes. Despite possessing extensive knowledge and experience in sports, transitioning into teaching roles exposes a gap in essential educational skills. This includes lesson planning, student assessment, classroom management, and effective communication with colleagues. One participant described: *"You come into this thinking your years on the field will translate easily into the classroom. But it's a whole different ball game. Suddenly, you're expected to draft lesson plans, evaluate students, and juggle administrative tasks. It's like, I know how to coach for the win, but how do I grade a paper? How do I tailor a lesson to a student…?"(S8: Kai)*.

###### 3.1.4.1.2 Challenges to collaborative capabilities

Another challenging aspect is adapting to professional relationships within educational settings. Working collaboratively with teaching staff and administrators requires a shift in communication and interpersonal skills, different from those cultivated on the field or in the locker room. *"And then there's working with others — the teachers, the admin staff. It's a team effort, but the dynamics are so different. Learning to navigate those relationships is a whole lesson in itself."(W7: Yue)*.This challenge underscores the gap between the skills and qualifications they have and those required in their new roles. It highlights both the individual and systemic obstacles encountered in this transition.

##### 3.1.4.2 Psychological and identity-related adjustments

The transition of retired athletes into the higher education sector is not solely a professional journey but also a profound psychological and identity-related adjustment. This theme highlights the internal challenges individuals face as they navigate this significant life transition.

###### 3.1.4.2.1 Challenges in psychological and physical adjustment

Participants articulated the challenges in adjusting to a life that no longer revolves around the rigorous physical and psychological demands of professional sports. One reflection captures this struggle: *“… like stepping into a different world. Suddenly, the adrenaline, the routine, the physical exertion that defined my existence was gone. It was disorienting…”(W4:Wei)*. Another aspect of this is the psychological adjustment to a less physically demanding lifestyle, which can be as daunting as the professional transition. *“And it’s not just the physical side of things; it’s the mental game too…finding a new kind of fulfilment in academia, one that does not come from physical exertion requires a significant mental shift.”(W6: Rong)*.

###### 3.1.4.2.2 Lack of professional identity and satisfaction

A critical aspect of this transition is the reformation of professional identity. For many, their identity as athletes was a major source of personal and professional satisfaction. Moving away from this identity can lead to feelings of loss and uncertainty. *"It's strange, you know? One day you're this athlete with a clear sense of identity and purpose, and the next, you're trying to find your place in a different field…"(S1: Tan). So,* the lack of a clear professional identity in the academic world can lead to challenges in finding the same satisfaction and fulfillment that sports once provided.This theme reveals the deeply personal and often challenging aspects of retired athletes’ transition into the higher education sector. The journey involves not only acquiring new skills and knowledge but also undergoing significant psychological and identity transformations. These adjustments are critical for achieving a successful and satisfying second career in academia. The reflections shared by participants underscore the importance of support and guidance in navigating this complex transition.

### 3.2 Phase 2: Delphi study

The Delphi method was used to gather opinions from experts regarding the significant hurdles that retired athletes face when transitioning into teaching roles in higher education. The research findings were obtained through a three-phase data collection and analysis process. Out of the 25 experts who were invited to participate in the study, 17 took part in all three rounds (response rate = 68%).

#### 3.2.1 Round 1

Out of the 25 experts who were invited to participate in the study, 21 took part in the first round (response rate 84%). In this initial round, the experts responded to an open-ended question regarding the hurdles faced by retired athletes when applying for teaching roles in higher education. Their responses yielded 102 statements, including short narratives and single statements, which were then subjected to an inductive content analysis. This analysis produced a list of initial themes identified by the experts. Following this, the initial themes (ITA) identified from the thematic analysis were presented to the experts to gather their initial reactions and additional insights. This feedback was used to refine and expand the list of initial themes, resulting in 27 sub-categories that were used in the second Delphi round. Detailed information on sub-categories and changes across rounds:

Initial sub-categories derived: 27.
Examples of initial sub-categories:
Lack of formal teaching qualifications.
Limited knowledge of subject-specific pedagogy.
Challenges in adapting to academic culture.

#### 3.2.2 Round 2

In the second round of the study, the finalized list of 27 sub-categories was sent to the 21 experts who participated in the first round; 19 of them responded (response rate 90%). The responses were analyzed to assess the degree of homogeneity and convergence among the experts’ opinions. Sub-categories with a mode of 3 or greater were retained, eliminating 5 sub-categories that did not meet this criterion. The weightings and coefficients were calculated using the following formula:


$$w=\frac{\sum_{i=1}^{n}s_{i}}{N}$$

where:

*W*: is the weighting of each sub-category,
*S_i_*: is the score given by each expert,
*N*: is the total number of experts who responded.

Of the remaining 22 key themes, 13 reached homogeneity and convergence, while the remaining 9 sub-categories exhibited a standard deviation to the mean greater than or equal to 1. Consequently, these 9 sub-categories were included in the third round for further evaluation. Detailed information of sub-categories and changes across rounds (Table 4):

Sub-categories evaluated: 27.
Sub-categories retained: 22 (5 eliminated due to mode < 3).
Rationale for elimination: Sub-categories with lower relevance and consensus among experts.
Examples of retained sub-categories:
Difficulty in obtaining teaching certifications.
Insufficient training in educational methodologies.

**Table 4:** Themes and content of consensus from experts.

Themes	Content
Educational qualification	The experts unanimously recognized a prevalent gap in formal educational qualifications among retired athletes seeking teaching roles. This gap is not merely about possessing a degree but having specific academic credentials (the accredited teaching certifications) that are recognized and valued in higher educational contexts.
Limited literacy knowledge	The deficit in specific academic or pedagogical knowledge areas, particularly those essential for teaching at a higher education level, was identified as a significant barrier. This goes beyond basic literacy to encompass a comprehensive understanding of subject matter, pedagogical strategies, and the ability to engage critically with academic content.
Educational capabilities	Acknowledging the need for development in teaching methodologies and classroom management, skills underscored broader barriers in transitioning from an athletic to an academic environment. This dimension points to the essential nature of pedagogical skills that ensure effective teaching and learning, including curriculum design, assessment strategies, and the ability to foster inclusive, engaging learning environments.
Lack of systemic support and legal safeguards	Experts emphasized the broader institutional and policy-level difficulties that retired athletes face, highlighting a systemic lack of support and inadequate legal safeguards that hinder successful transitions.

#### 3.2.3 Round 3

In the third round, each of the 19 experts received a survey containing the 9 sub-categories identified in the previous round. A total of 17 experts provided feedback in this round (response rate = 89.5%). Based on their input, a consensus was reached on these sub-categories. During the first two rounds, consensus was reached on 13 and 9 sub-categories, respectively. Through this process, the researchers conducted an inductive analysis to group the sub-categories into abstract themes that reflected the critical dimensions of the most significant hurdles retired athletes face when transitioning into teaching roles in higher education. These 22 sub-categories were distilled into four key dimensions: Educational Qualification, Limited Literacy Knowledge, Educational Capabilities, and Lack of Systemic Support and Legal Safeguards. Detailed information of sub-categories and changes across rounds:

Sub-categories re-evaluated: 9 (with high standard deviation).
Sub-categories reaching consensus: 9.
Examples of consensus sub-categories:
Need for ongoing professional development.
Lack of institutional support structures.

## 4 Discussion

This research explored the experiences of retired athletes transitioning into the higher education sector by focusing on their motivations, barriers, difficulties, and challenges. An inductive thematic analysis was employed in Phase 1 to gather in-depth insights directly from the athletes, while Phase 2 utilized the Delphi method to refine and validate these findings through expert consensus. The results from Phase 1 provided a foundational understanding of the athletes’ personal experiences and intrinsic motivations. In contrast, Phase 2 expanded on these findings by incorporating expert perspectives, thus ensuring a comprehensive and validated understanding. The findings from both phases not only complement each other but also provide a nuanced and holistic view of the transition process. This dual-phase approach highlights the complexity of the transition and underscores the need for supportive structures to facilitate smoother transitions for retired athletes into higher education roles.

### 4.1 Motivation

This research shows that motivation for retired athletes to transition into the higher education sector is multifaceted, encompassing passion and advocacy, personal identity, professional development, and financial considerations. Phase 1 demonstrates how these motivations are personal yet relevant to everyone in the group. The intrinsic desire to remain connected to sports while contributing to societal well-being reflects a commendable pursuit of purpose post-retirement, also serving as a bridge between their past athletic identities and their future professional selves. Silver (2021) emphasizes that pursuing an education career could help retired athletes redefine their professional identities while leveraging their passion. This passion-driven motivation is echoed in Phase 1, where participants articulated a strong desire to impact future generations’ health and fitness positively. Meanwhile, the challenge of personal identity reconstruction highlighted by Cosh et al. (2013) and Warriner and Lavallee (2008) resonates with the findings of Phase 1, suggesting a complex interplay between the loss of the athlete identity and the formation of a new professional identity within the academic sector. The literature and study findings suggest that the education sector provides a conducive environment for this identity transition, enabling retired athletes to rediscover purpose and fulfillment beyond their athletic careers.

Phase 2’s Delphi process further validated these motivational factors by achieving consensus among HR experts on the significance of these themes. Experts confirmed that passion, personal identity, professional development, and financial stability are critical motivators for retired athletes transitioning into higher education roles. This expert validation reinforces the Phase 1 findings and highlights the multifaceted nature of the motivation behind this career shift. In addition, Coffey and Davis (2019) highlight the importance of transferable skills for professional development in new careers, aligning with Phase 1’s findings that retired athletes bring valuable skills to the education sector. This emphasizes the mutual benefit of such transitions, not only for the athletes in terms of professional development and stability but also for the education sector, which gains from their unique perspectives and experiences. Finally, the financial aspect of motivation, as discussed by Barriopedro et al. (2018), and supported by the broader implications of policies (Zheng and Ying, 2016), underlines the practical considerations that retired athletes face when transitioning to the academic sector. This pursuit of financial stability and the appeal of academic careers for long-term security is consistent with Phase 1’s findings, where financial and stability considerations were significant motivators. Acknowledging these financial motivations complements the more intrinsic motivations of passion and identity, offering a comprehensive view of the factors driving this career transition.

These critical discussions highlight the importance of recognizing and supporting these motivational factors to facilitate smoother transitions for retired athletes into meaningful and fulfilling second careers in education. Future research and policy should thus focus on developing frameworks and interventions that address these motivational aspects, enhancing retired athletes’ contributions to the education sector and supporting their professional and personal development post-retirement.

### 4.2 Barriers

The lack of professional recognition and value of physical education as a critical field has been found in this research, which resonates with the discussion of Murray (2005) on the substantial adaptations required by individuals transitioning into teacher-educator roles, emphasizing the struggle to establish a new identity within the academic setting.

In Phase 1, retired athletes reported experiencing these barriers firsthand, highlighting the absence of explicit recognition processes and structures for physical education roles. This systemic undervaluation, as echoed by Barker et al. (2021), underscores the broader societal underestimations of physical education’s contribution to holistic education. Retired athletes face a dilemma in reshaping their self-concept beyond their sports careers, a barrier well-documented by Küettel et al. (2017). This barrier corresponds with our findings in Phase 1, where professional recognition and the importance of physical education emerged as notable barriers.

Phase 2, through the Delphi method, confirmed and refined these findings. The lack of institutional support and legal safeguards, as noted by Holden and Hansen (1989), was identified as a significant barrier by HR experts, indicating a systemic failure to facilitate smooth transitions for athletes into academic roles. This aligns with the feedback from experts in Phase 2, highlighting a systemic lack of support and inadequate legal safeguards that hinder successful transitions.

Furthermore, Bain and Gray (2018) emphasized that defining the identity of teacher educators before considering their professional development speaks volumes about the necessity for clarity in roles and expectations within the education sector. This need is acutely felt by retired athletes who, as highlighted in our findings, often face the undervaluation of their new roles. Our findings highlight that these individuals often experience an identity crisis, which is compounded by the societal and institutional failure to recognize the unique value and perspective that athletes bring to the educational landscape, particularly in physical education. Additionally, systemic and institutional barriers identified by Küettel et al. (2017) and Barker et al. (2021), such as the lack of supportive frameworks and the expectations of physical education roles, highlight the need for a paradigm shift. There is a pressing requirement for policies and practices that not only recognize the unique contributions of retired athletes but also actively support their transition into educational roles. The integration of findings from both phases of our study underscores the importance of developing supportive structures and recognizing the unique value that retired athletes can bring to the higher education sector.

### 4.3 Difficulties

We discovered that retired athletes face significant difficulties when transitioning to the higher education sector, such as adapting to the academic environment and aligning their qualifications with academic requirements. The literature highlights the critical role that high-quality academic programs and educational standards play in field the higher education (Ali et al., 2018), which sets a high bar for entry into academic roles, where retired athletes often find a mismatch between their qualifications and the expectations of higher education institutions. The findings of Phase 1 and Phase 2 support this literature and highlight the difficulties faced by retired athletes due to their often-non-traditional educational background. Also, the importance of acquiring the necessary skills and qualifications for successful career transitions, as noted by de Subijana et al. (2020), resonates with the difficulties identified in both phases. This aligns with the broader observation that athletes with lower educational levels may struggle with employability, underscoring the need for targeted interventions to bridge this qualifications gap. This gap not only affects the employability of retired athletes but also has broader implications for their inclusion in society and their mental health (Gouttebarge, 2016; Goulart et al., 2021).

Furthermore, the literature further illuminates the difficulties of adapting to the educational environment, emphasizing the necessity of embracing a continuous learning mindset (Holme, 2016), which is a critical aspect of this adaptation process. This adaptation is multifaceted, requiring not only the acquisition of new knowledge and pedagogical skills but also a significant transformation in the athletes’ approach to learning and identity. The insights from Phase 1 and Phase 2 echo these findings. In Phase 1, retired athletes described their struggles with adapting their teaching methods and engaging with the diverse needs of students in higher education. Phase 2 further validated these difficulties, as HR experts highlighted the same difficulties and emphasized the need for professional development programs tailored to this unique population. The findings also highlight systemic and personal difficulties that this population faces. To facilitate these transitions, targeted support mechanisms and policy interventions are needed. These should focus on developing educational qualifications and aiding the adaptation process within the educational environment. By addressing these difficulties, we can better support retired athletes in their transition to meaningful and fulfilling roles in higher education.

### 4.4 Challenges

Two significant challenges emerged from this research: a lack of teaching experience and collaborative capabilities with teaching staff and administrators. Regarding lack of teaching experience, both the literature and Phase 1 identified a critical gap in formal teaching experience and pedagogical skills among retired athletes. This aligns with Payne et al. (2017) and Till et al. (2022), who mentioned that retired athletes may face challenges in developing the necessary pedagogical skills to excel academically. Meanwhile, Phase 1’s findings reveal retired athletes’ struggles with developing essential educational skills, a feeling echoed by experts in Phase 2, who recognize the pressing need for development in teaching methodologies and classroom management skills. This consensus underscores the importance of implementing targeted professional development initiatives that equip retired athletes with the requisite pedagogical competencies for academic success. Payne et al. (2017) stressed that professional development programs tailored to address these specific needs can help retired athletes acquire essential teaching skills and enhance their effectiveness in the classroom.

Moreover, the challenges involved in transitioning to collaborative academic environments, which were identified in Phase 1, have been supported by existing literature. Al-Baadani and Abbas (2020) noted that developing strong collaborative skills takes time and is essential for building relationships with colleagues, navigating departmental dynamics, and aligning teaching approaches with institutional goals. Phase 2 findings confirmed that retired athletes may struggle to fit into the collaborative academic environment due to a lack of experience in academic teamwork. To address this issue, programs that foster collaborative skills are needed to help retired athletes effectively navigate departmental dynamics and align their teaching approaches with institutional goals, ultimately contributing positively to the educational environment.

More importantly, retired athletes face significant psychological and identity-related adjustments during their transition. Phase 1 participants have expressed challenges in adapting to a life outside the professional sports industry, which is consistent with the findings of Dimoula (2013), who noted that athletes often experience stress, anxiety, and a sense of loss when they lose their athletic role and solid athletic identity. Factors such as family support, coaching, and the sports club environment can play a significant role in helping athletes navigate this transition (Franck and Stambulova, 2018). Also, planning for retirement and considering areas such as studies, family, and leisure can also influence the challenges athletes face post-retirement (Barriopedro et al., 2019). Additionally, athletes often develop a strong sense of identity tied to their sports careers, making it challenging to establish a new professional identity outside the sports realm (Lavallee, 2005). As discussed by Aston et al. (2022), the shift from being a competitive athlete to an educator may lead to feelings of uncertainty, loss of purpose, and a diminished sense of self-worth. Therefore, the absence of a clear professional identity and the perceived lack of fulfillment in their new roles can contribute to dissatisfaction and emotional distress among retired athletes transitioning into teaching positions in higher education.

The complementary insights from Phases 1 and 2 underscore the complexity of these challenges and highlight the need for targeted interventions and supportive structures to facilitate smoother transitions for retired athletes into the higher education sector.

## 5 Limitations and future directions

Although this study made valuable contributions, it had some limitations. Firstly, the study focused solely on retired athletes in the Chinese context. The experiences of these individuals are highly individualistic and influenced by numerous factors, such as cultural, socio-economic, and institutional socioeconomic may not have captured all these factors in their entirety. Additionally, the focus on retired athletes transitioning specifically into the higher education sector may overlook broader career pathways and transitions experienced by this group. The study’s findings are also predicated on the assumption of a successful transition, potentially omitting the experiences of those who had unsuccessful attempts at transitioning.

Another limitation of our research relates to the use of member (i.e., participant) checking in the thematic analysis coding and theme generation process. Specifically, Smith and McGannon (2018) argued that member checking of thematic analysis findings might not necessarily be helpful, as participants may be unable to remain objective when reviewing their own experiences. We agree and acknowledge this potential limitation. Nevertheless, we would like to ensure that we implemented member checking mainly at the transcription and coding levels rather than at the interpretation of findings. As outlined by Harper and Cole (2012), member checking plays a crucial role in making participants feel valued and ensures the accuracy of the transcription process, thus contributing positively to the reliability of our research. Therefore, this practice was intended to mitigate concerns regarding the lack of participants’ research expertise while enhancing the trustworthiness of the data.

Moreover, quantitative research methods could complement the qualitative insights provided by this study, offering broader generalizability and the ability to identify trends and patterns in the transition experiences of retired athletes. Such studies could explore the impact of specific factors, such as the role of mental health support, the effectiveness of professional development programs, and the influence of institutional policies on the success of these transitions.

Additionally, exploring the perspectives of other stakeholders in the higher education sector, such as administrators, students, and fellow academics, would also provide a more holistic understanding of the impact of retired athletes on the academic community and the factors that facilitate or hinder their integration and success. Also, we recognize the importance of identifying both facilitating and impeding factors that affect the transition of retired athletes into the higher education sector. Facilitators such as mentorship programs, institutional support systems, flexible academic scheduling, and professional development opportunities can significantly inform future practical works and support successful transitions.

Future research should first aim to incorporate both facilitating and impeding factors to offer a more comprehensive understanding of this transition process. Secondly, future research should also explore the effectiveness of dual career programs initiated by sports organizations and their impact on the post-retirement transitions of athletes. Assessing the long-term outcomes of such initiatives can provide valuable insights into how sports organizations can enhance support structures and collaborate with educational sectors to foster more inclusive and flexible pathways for athletes with non-traditional educational backgrounds. Addressing these areas in future research can enrich our understanding of retired athletes’ career transitions, contributing to more inclusive, diverse, and enriching academic environments.

## 6 Conclusion

This study has provided a comprehensive exploration of the psychosocial factors influencing retired athletes’ transition into the higher education sector. Our research, spanning both qualitative interviews and a Delphi method, has uncovered significant insights into the unique motivations, barriers, difficulties, and challenges that retired athletes encounter during this pivotal career shift. Key findings highlight that while the motivation to transition is driven by a mix of passion for education and personal development, financial stability, and a desire to maintain a connection to sports, these athletes face substantial barriers, including lack of professional recognition, difficulties in aligning their past experiences with academic requirements, and the challenges of acquiring new pedagogical skills. The study notably emphasizes the complex interplay of these factors, which affect not only the individual’s ability to transition but also the quality of their integration into the academic environment. Further, our findings suggest that supportive structures and targeted interventions are crucial in facilitating smoother transitions for retired athletes. The presence of clear recognition processes, professional development programs, and supportive policies can significantly mitigate the challenges faced by these individuals. Institutions that harness the unique perspectives and skills of retired athletes can enrich their educational offerings, thereby fostering a more inclusive and diverse academic community. In moving forward, it is vital for policy efforts and institutional strategies to consider these factors comprehensively to enhance retired athletes’ successful integration into the higher education sector. Future research should continue to explore these psychosocial influencers, extending beyond the education sector to other career domains to which retired athletes might transition.

## Data availability statement

The raw data supporting the conclusions of this article will be made available by the authors, without undue reservation.

## Ethics statement

The studies involving humans were approved by the Research Ethics Committee of Hunan University of Technology. The studies were conducted in accordance with the local legislation and institutional requirements. The participants provided their written informed consent to participate in this study.

## Author contributions

YZ: Supervision, Validation, Writing – original draft, Writing – review & editing, Data curation, Formal analysis, Funding acquisition, Investigation, Methodology, Resources. ZZ: Conceptualization, Formal analysis, Investigation, Methodology, Project administration, Resources, Writing – review & editing, Writing – original draft.
