# Supplementary material for: From athletic excellence to academic influence: a study of retired Chinese athletes transitioning into the higher education sector
Source: Front Psychol. 2024 Jun 18;15:1401575. doi: 10.3389/fpsyg.2024.1401575 (PMC11217518; doi:10.3389/fpsyg.2024.1401575)
Supplement: Supplementary file 1 [file Table_1.DOCX]

For a participant who is currently unemployed but is expected to transition into the higher education sector:

1. Preparation and Expectations:

"As you prepare to transition into the higher education sector, what steps are you taking to equip yourself for this new role? What are your expectations for working in this sector?"

1. Motivations for Transition:

"What motivates you to pursue a career in the higher education sector after your athletic career? How do you think your background as an athlete will influence your approach to this new role?"

1. Anticipated Challenges:

"What challenges do you anticipate facing as you transition into the higher education sector? How do you plan to address or overcome these challenges?"

1. Skills and Adaptations:

"Considering your skills and experiences as an athlete, which of these do you believe will be most beneficial in your future role in higher education? Are there any new skills you feel you need to develop?"

1. Long-term Vision:

"What is your long-term vision for your career in the higher education sector? How do you hope to impact the field and the students you will work with?"

For a participant who is currently working as a teacher in higher education:

1. Reflecting on Your Journey:

"Can you describe your journey from being a professional athlete to your current role in the higher education sector? What were some of the pivotal moments or decisions that influenced your transition?"

1. Motivations and Aspirations:

"What motivated you to pursue a career in the higher education sector after retirement from professional sports? How do your aspirations in this sector align with your experiences as an athlete?"

1. Overcoming Challenges:

"What have been some of the most significant challenges you've faced in adapting to your role in higher education? How have you addressed these challenges?"

1. Skills and Contributions:

"How do you believe your skills and experiences as a professional athlete contribute to your current role? Are there any unique perspectives or approaches you bring to your work in higher education?"

1. Future Outlook:

"Looking ahead, what are your goals or ambitions within the higher education sector? How do you see your role evolving, and what impact do you hope to have on your students and the broader community?"

The interview questions to target leaders in the human resources department in higher education:

1. From your experience in human resources and the recruitment of retired athletes for teaching positions, what do you believe are the most significant barriers these candidates face during the transition?
2. In your recruitment process, how do you assess the specific academic or pedagogical knowledge of retired athletes? What challenges have you observed in this area, and how are they mitigated?
